# Optimization of Lyophilized Hyperacute Serum (HAS) as a Regenerative Therapeutic in Osteoarthritis

**DOI:** 10.3390/ijms22147496

**Published:** 2021-07-13

**Authors:** Isabel Olmos Calvo, Olga Kuten-Pella, Karina Kramer, Ágnes Madár, Szilvia Takács, Dorottya Kardos, Diána Simon, Szabina Erdö-Bonyár, Timea Berki, Andrea De Luna, Stefan Nehrer, Zsombor Lacza

**Affiliations:** 1 OrthoSera GmbH, Dr. Karl-Dorrek-Straße 23–29, 3500 Krems an der Donau, Austria; olga.kuten@orthosera.com (O.K.-P.); agnes.madar@orthosera.com (Á.M.); szilvia.takacs@orthosera.com (S.T.); 2 Center for Regenerative Medicine, Danube University of Krems, 3500 Krems an der Donau, Austria; karina.kramer@donau-uni.ac.at (K.K.); Andrea.DeLuna@donau-uni.ac.at (A.D.L.); stefan.nehrer@donau-uni.ac.at (S.N.); 3 Research Center for Natural Sciences, 1117 Budapest, Hungary; dorottya333@gmail.com; 4 Department of Immunology and Biotechnology, Medical School, University of Pécs, 7624 Pécs, Hungary; simon.diana@pte.hu (D.S.); erdo-bonyar.szabina@pte.hu (S.E.-B.); berki.timea@pte.hu (T.B.); 5 Department of Sport Physiology, University of Physical Education, 1123 Budapest, Hungary; zsombor.lacza@orthosera.com; 6 Institute of Translational Medicine, Semmelweis University, 1085 Budapest, Hungary

**Keywords:** hyperacute serum, osteoarthritis, joint regeneration, explant co-culture, blood derived product, inflammatory cytokines, bone remodeling

## Abstract

Hyperacute serum (HAS) is a blood derivative product that promotes the proliferation of various cell types and controls inflammation in vitro. The aim of this study is to investigate the regenerative potential of different formulations of HAS, including lyophilized and hyaluronic acid combined versions, to obtain a stable and standardized therapeutic in osteoarthritis (OA), which may be able to overcome the variability limitations of platelet-rich plasma (PRP). Primary human osteoarthritic chondrocytes were used for testing cellular viability and gene expression of OA-related genes. Moreover, a co-culture of human explants of cartilage, bone and synovium under inflammatory conditions was used for investigating the inflammatory control capacities of the different therapeutics. In this study, one formulation of lyophilized HAS achieved the high cell viability rates of liquid HAS and PRP. Gene expression analysis showed that HAS induced higher *Col1a1* expression than PRP. Cytokine quantification from supernatant fluids revealed that HAS treatment of inflamed co-cultures significantly reduced levels of IL-5, IL-15, IL-2, TNFα, IL-7 and IL-12. To conclude, lyophilized HAS is a stable and standardized therapeutic with high potential in joint regeneration.

## 1. Introduction

Osteoarthritis (OA) is the most common joint degenerative disease, especially affecting the elderly population, which it is expected to increase in the next decades [1]. OA is characterized by articular cartilage and subchondral bone destruction leading to locomotor disability and pain. The lack of an effective current treatment to regenerate or impede joint destruction in the context of OA has left alleviating symptoms as the only therapeutic option until the joint is surgically replaced [2]. 

Different blood derivate products obtained after whole blood centrifugation has become a novel and attractive therapy [3]. The aim is to isolate and concentrate specific components that may have a potential effect in the context of tissue engineering. Different preparation processes lead to different compositions and therefore, different potential outcomes in modulating cell proliferation and inflammation [4]. These blood derivative products are then intraarticularly injected into osteoarthritic joints. Particularly, platelet-rich plasma (PRP) has become the therapeutic mostly used for this purpose [5]. However, there are multiple studies that have shown controversy about its benefits and it may not be the optimal solution to stop or reverse the degenerative process in the joints [6,7,8]. The main disadvantage relates to its composition variability due to different production techniques and its relevant content of pro-inflammatory agents such as fibrin and leukocytes [9,10,11,12,13]. 

In contrast, hyperacute serum (HAS) is a cell and fibrin-free solution, obtained after pressing out fibrin clots, which maintains the highly regenerative capacities of PRP. Previous studies of HAS used the freshly isolated formulation, a liquid version, which proved to induce cell proliferation of chondrocytes, bone marrow mesenchymal stem cells, osteoblasts in cell culture and in a bone ischemic model [14,15,16,17,18]. Kardos et al. established a more complex biological model consisting of a human explant co-culture of bone, articular cartilage and synovium, which was used to study the effect of HAS under inflammatory conditions showing that HAS reduced inflammation as well as induced cell proliferation [19]. This human explant model allows communication between tissues, better representing the damage caused during OA within the joint, which experimentally is sometimes simplified to cartilage or bone in vitro cultures. It is known that OA also causes synovitis, effusion of synovial fluid, osteophyte formation and muscle weakness and atrophy, therefore supporting the use of more complex models [20,21,22]. In fact, synovial fluid has been used to identify and quantify possible biomarkers of OA, including the pro-inflammatory interleukins IL-6, IL-1β and tumor necrosis factor alpha (TNFα); however, the complexity and high amount of proteins involved in OA, together with the variability between patients has not so far allowed to reach a clear conclusion about this issue [23,24,25,26]. Still, inflammation during OA has been shown to have an impact on extracellular matrix (ECM) composition, perpetuating the pathology [27].

Freshly isolated HAS enables autologous therapy but has limitations during clinical administration. In order to produce a stable and standardized product that allows for long storage periods, HAS in freeze dried format has been produced and tested. During the lyophilization process, sublimation of the contained frozen water and the subsequent separation of vapor produces a powder—in our case, HAS powder [28,29]. Lyophilization may allow to use the product in an allogenic way; the idea is to produce it by pooling the serum from fibrin clots of multiple healthy and young donors, reducing variability between batches, and providing a nutrient-enriched formulation. For this purpose, two versions of lyophilized HAS were produced: the first one with an extra immunoglobulin M (IgM) filtration step, referred to as “filtered lyophilized HAS”; and a second one without the mentioned step, “non-filtered lyophilized HAS”. Moreover, visco-supplementation of hyaluronic acid (HA) has been widely used as a treatment of OA due to its mechanical properties and its demonstrated capacity for reducing local inflammation [7,30]. The combination of HA with a therapeutic that may induce a synergistic positive biological effect has gained interest in the last years [31,32,33]. The aim of this study was to test if lyophilized HAS reaches the regenerative potential of other blood derivative products for OA treatment alone and in combination with HA.

## 2. Results

### 2.1. Optimizing HAS Production Based on Chondrogenic Viability Effect

**Figure 1:**
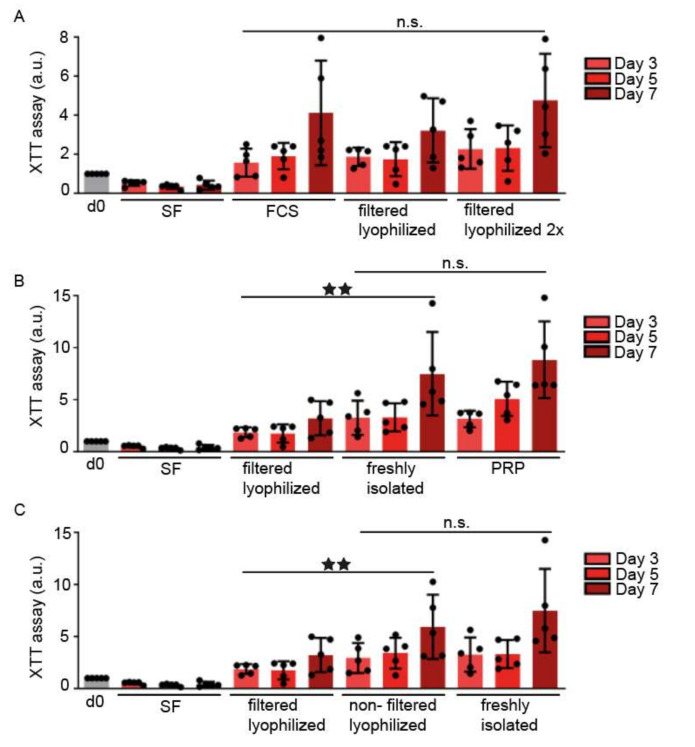
Chondrocyte viability by XTT assay over time (days 3, 5 and 7). Comparisons include (**A**) two concentrations (10% and 20%) of filtered lyophilized HAS (represented as filtered lyophilized and filtered lyophilized 2×, respectively) and 10% FCS; (**B**) filtered lyophilized HAS, freshly isolated HAS and PRP; (**C**) the two versions of lyophilized (filtered and non-filtered) and freshly isolated HAS. Serum free (SF) and day 0 (d0) were used as negative controls in all experiments (n = 5). Bars represent arithmetic means and error bars show standard deviations (SD). Other abbreviations: not significant (n.s.), arbitrary units (a.u.), fetal calf serum (FCS), level of significance where *p* < 0.01 (**).

Two concentrations of filtered lyophilized HAS were compared to the known gold standard supplementation, fetal calf serum (FCS) (Figure 1A). Statistical time-dependent analysis showed a significant increase in cell viability over time after supplementation with 10% FCS and 20% filtered lyophilized HAS (*p* = 0.0298, *p* = 0.0375, respectively). When linear regressions of the three groups were compared, no significant differences between the groups were found. This data suggests that in vitro chondrocyte viability after filtered lyophilized HAS supplementation reaches an FCS-induced effect. Moreover, doubling the concentration of lyophilized HAS to 20% of the total volume did not increase chondrocyte viability. Then, filtered lyophilized HAS was compared to freshly isolated HAS and PRP (Figure 1B). Freshly isolated HAS and PRP significantly increased chondrocyte viability over time (*p* = 0.029 and *p* = 0.002, respectively) and their linear regression comparison showed no statistical difference. However, filtered lyophilized HAS did not support chondrocyte viability as strongly as freshly isolated HAS and PRP (*p* = 0.0047 and *p*-value not possible to calculate due to the high difference of the linear slopes, respectively). The potential removal of nutrient supply within filtered lyophilized HAS, due to the IgM filtration step, led us to produce a new version of lyophilized HAS which excluded the already mentioned filtration step. The same experiment was repeated with this new HAS formulation, demonstrating that non-filtered lyophilized HAS increased chondrocyte viability over time (*p* = 0.0445). Linear regression comparisons between non-filtered lyophilized and freshly isolated HAS revealed no statistical difference (Figure 1C). In contrast, same comparison showed that non-filtered lyophilized HAS significantly promoted cell viability when compared to the filtered version of HAS (*p* = 0.0075). This data suggests that non-filtered HAS achieves the outstanding supplementation levels of the freshly isolated formulation, with the advantages of being a lyophilized powder. 

To investigate a possible synergistic effect between HA and HAS, HA was supplemented to the filtered and non-filtered lyophilized HAS formulations. Cell viability measurements via XTT assay showed no statistical significance (Table 1).

### 2.2. Gene Expression of Osteoarthritis-Related Genes after Blood Derivatives Supplementation

**Figure 2:**
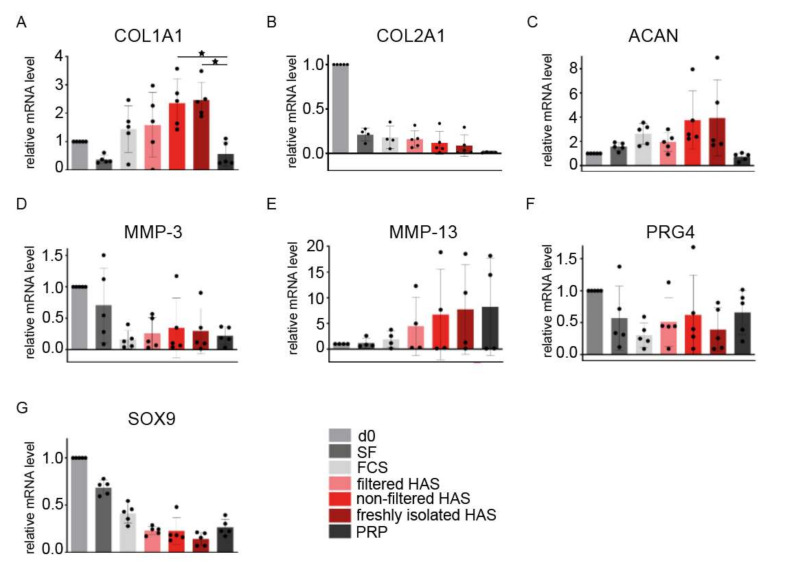
Gene expression of OA-related genes from a chondrogenic 2D culture after supplementation with different versions of HAS and PRP (n = 5). Tested genes include (**A**) *Col1a1*, (**B**) *Col2a1*, (**C**) *Acan*, (**D**) *Mmp-3*, (**E**) *Mmp-13*, (**F**) *Prg4*, and (**G**) *Sox9*. Bars represent arithmetic means and error bars, SD. Abbreviations: day 0 (d0), serum free (SF), fetal calf serum (FCS), hyperacute serum (HAS), platelet-rich plasma (PRP), collagen type I alpha 1 chain (COL1A1), collagen type II alpha 1 chain (COL2A1), aggrecan (ACAN), metalloproteinases (MMP), proteoglycan 4/lubricin (PRG4) and SRY-box transcription factor 9 (SOX9). Level of significance where *p* < 0.05 (*).

Gene expression analysis showed that genes related to ECM formation such as collagen type I alpha 1 chain (*Col1a1*), collagen type II alpha 1 chain (*Col2a1*) and aggrecan (*Acan*) were upregulated after supplementation with the different HAS formulations when compared to PRP (Figure 2A–C). However, only PRP expression for *Col1a1* was significantly lower when compared to non-filtered lyophilized and freshly isolated HAS (*p* = 0.04 and *p* = 0.034, respectively). Statistical tests from *Col2a1* and *Acan* data did not reach statistical significance, probably due to donor variability. *Col2a1* expression decreased for all conditions when compared to day 0, similarly to the SRY-Box transcription factor 9 (*Sox9)* expression (Figure 2B,G). Metalloproteinases *Mmp-3*, *Mmp-13* and lubricin (*Prg4)* expression showed high variability between patients; consequently, no conclusive outcome could be defined (Figure 2D–F). 

The same gene expression analysis was performed on the samples where HAS was combined to HA (Table 2). The results showed no significant difference for any tested gene.

### 2.3. Protein Profiling after Treating the Inflamed Explant Co-Culture Model with HAS

**Figure 3:**
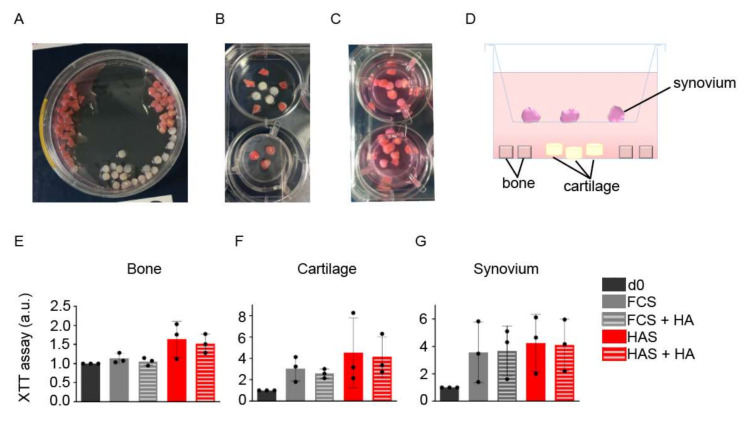
Establishment of an inflammatory ex vivo joint model. (**A**) Bone, cartilage, and synovium fragments from human samples of patients undergoing knee replacement were minced and transferred to cultured wells. (**B**,**C**) In each well, five fragments of bone, four of cartilage and two or three of synovium were placed as shown in the images and (**D**) in the side view scheme. XTT viability assay after a total of 7 days of culture was performed for (**E**) bone, (**F**) cartilage and (**G**) synovium. Bars represents arithmetic mean values ± SD (n = 3). Abbreviations: day 0 (d0), serum free (SF), fetal calf serum (FCS), hyperacute serum (HAS), platelet-rich plasma (PRP), hyaluronic acid (HA), arbitrary units (a.u.).

Human fragments of bone, articular cartilage and synovial tissues were used to establish a co-culture mimicking inter-tissue communication located at the joints (Figure 3A–D). After 2 days under IL-1β stimulation and a subsequent period of 5 days in the presence of non-filtered HAS and/or HA, XTT viability tests were performed. A one-way ANOVA test showed no significant difference between the groups in all tissues (Figure 3E–G). 

**Figure 4:**
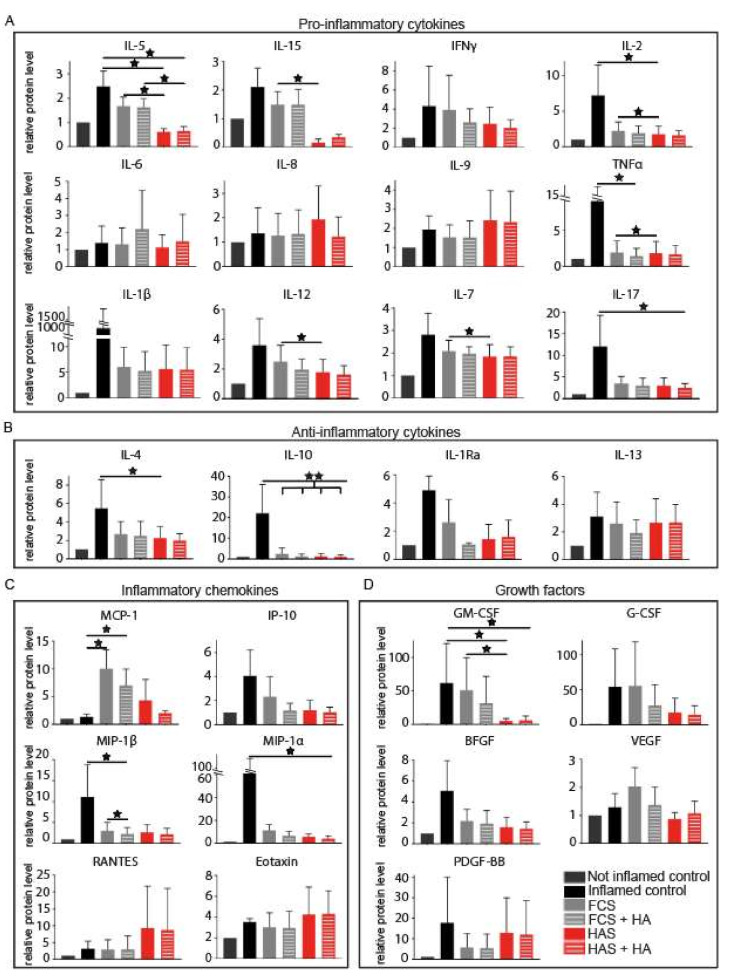
Quantification of cytokines and growth factors from supernatant fluids after 5 days of treatment with HAS and/or HA of a previously inflamed explant human co-culture. Proteins were grouped as (**A**) pro-inflammatory cytokines, (**B**) anti-inflammatory cytokines, (**C**) inflammatory chemokines and (**D**) growth factors. Inflamed (positive) control correspond to samples exposed to inflammation, before any treatment was added at day 0. Not inflamed (negative) control represent samples that were not exposed to inflammatory conditions before day 0. Bars represent arithmetic mean values ± SD (n = 3). Abbreviations: fetal calf serum (FCS), hyperacute serum (HAS), hyaluronic acid (HA), interleukins (IL-), tumor necrosis factor alpha (TNFα), interferon gamma (IFNγ), interleukin 1 receptor antagonist (IL-1Ra), monocyte chemotactic protein 1 (MCP-1), 10 KDa interferon gamma-induced protein (IP-10), macrophage inflammatory proteins (MIP), regulated upon activation normally T-expressed and presumably secreted (RANTES), granulocyte macrophage colony stimulating factor (GM-CSF), granulocyte colony stimulating factor (G-CSF), vascular endothelial growth factor (VEGF), basic fibroblast growth factor (BFGF) and platelet-derived growth factor subunit B (PDGF-BB). Level of significance where *p* < 0.05 (*) and *p* < 0.01 (**).

At the same time point, supernatant fluids were collected, and cytokines and growth factors were quantified (Figure 4). When compared to FCS, HAS supplementation induced a significant decrease of the pro-inflammatory cytokines IL-5 (*p* = 0.021), IL-15 (*p* = 0.045), TNFα (*p* = 0.038), IL-2 (*p* = 0.034), IL-7 (*p* = 0.031) and IL-12 (*p* = 0.048); and the growth factor GM-CSF (*p* = 0.045). HA induced a significant reduction of MIP-1β expression when compared to FCS alone (*p* = 0.045). The combination of HAS and HA only maintained the significantly different expression for IL-5 (*p* = 0.021). 

Moreover, the presence of multiple proteins including IL-2, TNFα, IL-17, IL-1β, IL-15, IL-7, IL-12, IL-5, IL-4, IL-10, IL-1Ra, IP-10, MIP-1β, MIP-1α and BFGF decreased in all groups when the inflammatory stimuli was removed; however, only, IL-10 decreased significantly for all conditions (*p* = 0.009). When compared to the positive control, HAS reduced the amount of IL-4, IL-5, GM-CSF and IL-2 found in the supernatant fluids (*p* = 0.039, *p* = 0.018, *p* = 0.018 and *p* = 0.036, respectively); HA reduced TNFα (*p* = 0.039) and MIP-1β (*p* = 0.018); HAS and HA combined influenced IL-17 (*p* = 0.039), IL-5 (*p* = 0.024), GM-CSF (*p* = 0.039) and MIP-1α (*p* = 0.039). Interestingly, after removing the inflammatory stimulation, MCP-1 increased for the conditions containing FCS alone and in combination with HA (*p* = 0.018 and *p* = 0.039, respectively), but not for the ones containing HAS. HAS supplementation with and without HA showed a non-significant trend in increasing the levels of RANTES and Eotaxin. All *p*-values are listed in Supplementary Materials (Table S1).

**Figure 5:**
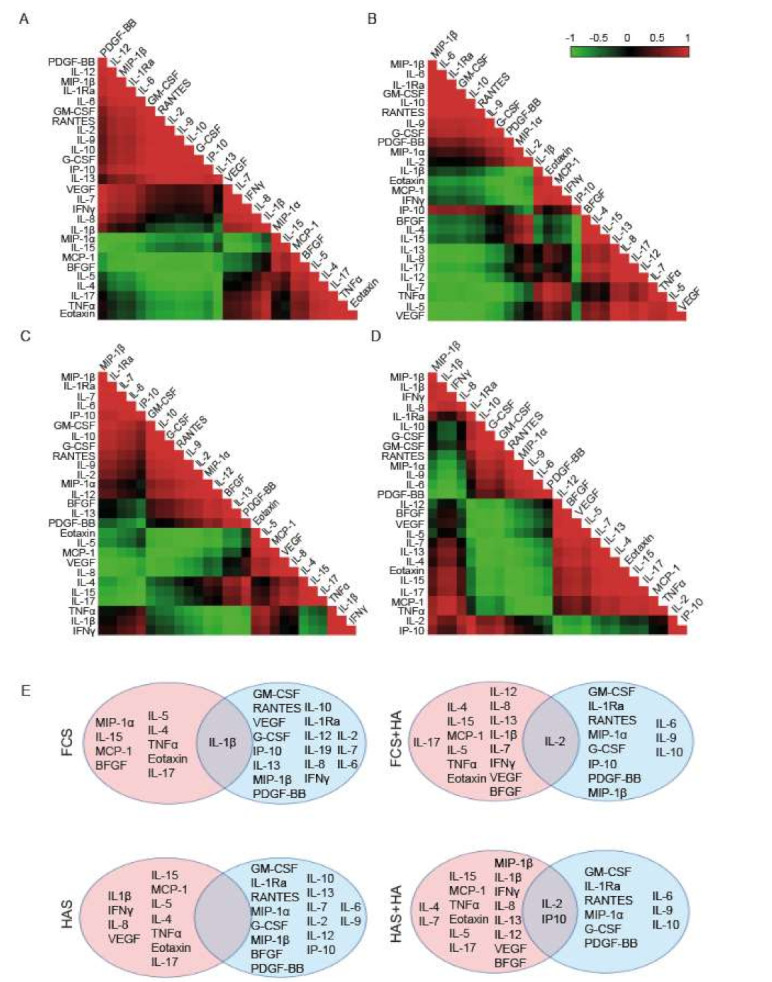
Correlations of cytokines and growth factor changes visualized as heatmaps for all conditions: (**A**) FCS, (**B**) FCS + HA, (**C**) HAS and (**D**) HAS + HA. Red color represents values close to 1 of the Pearson’s correlation, suggesting positive correlation patterns. Green color represents values for the Pearson’s correlation close to −1, indicating negative correlations. Black color means no direct correlation between the genes. (**E**) Scheme of genes manually separated according to their correlations into two blocks that negatively correlate between them. There is no color code meaning. (N = 3). Abbreviations: fetal calf serum (FCS), hyperacute serum (HAS), hyaluronic acid (HA), interleukins (IL-), tumor necrosis factor alpha (TNFα), interferon gamma (IFNγ), interleukin 1 receptor antagonist (IL-1Ra), monocyte chemotactic protein 1 (MCP-1), 10 KDa interferon gamma-induced protein (IP-10), macrophage inflammatory proteins (MIP), regulated upon activation normally T-expressed and presumably secreted (RANTES), granulocyte macrophage colony stimulating factor (GM-CSF), granulocyte colony stimulating factor (G-CSF), vascular endothelial growth factor (VEGF), basic fibroblast growth factor (BFGF) and platelet-derived growth factor subunit B (PDGF-BB).

Correlations of protein changes after supplementation of the four conditions: FCS or HAS ± HA ((day 0, inflamed control)—(day 5, treatment)) were calculated. Pearson’s correlations were very strong between the groups where HA was added, and their controls: FCS or HAS alone (r = 0.988 and r = 0.984, respectively). However, the correlation between HAS and FCS was weaker (r = 0.649), suggesting a more different cytokine and growth factor profile. Individual gene expressions were analyzed, correlated, and plotted as heatmaps for FCS (Figure 5A), FCS+HA (Figure 5B), HAS (Figure 5C) and HAS+HA (Figure 5D). Values for each correlation can be found in Supplementary Table S2 (Table S2). When analyzing individual genes, the FCS-supplemented group strongly correlated genes gathered as follows: PDGF-BB, IL-12, MIP-1β, IL1-Ra and IL-6; GM-CSF, RANTES, IL-2, IL-9, IL-10, G-CSF, IP-10 and IL-13; VEGF, IL-7, IFNγ, IL-8, and IL-1β; MIP-1α, IL-15, MCP-1 and BFGF. This last subgroup correlated negatively to the two first clusters listed. Lastly, the strongly correlated cluster IL-5, IL-4, IL-17, TNFα and Eotaxin negatively correlated to most of the genes grouped in the second cluster. The group treated with HAS showed that strongly correlated genes tended to form smaller clusters, leading to less complex negative correlations. For example, the cluster MIP-1β, IL-1Ra, IL-7 and IL-6 negatively correlated to the subgroups Eotaxin, IL-5, and MCP-1; VEGF and IL-8; and IL-4, IL-15 and IL-17. The strongly correlated subgroup formed by GM-CSF, IL-10, G-CSF, RANTES, IL-9, IL-2, MIP-1α and IL-12 negatively correlated to the clusters TNFα, IFNγ and IL-1β; and Eotaxin, IL-5, MCP-1, VEGF and IL-8.

To better understand differences between HAS and FCS-supplemented cultures, and the possible synergistic effect of HA to HAS, proteins were divided into two blocks depending on their positive or negative correlations (Figure 5E). This approach showed that although interactions between cytokines and growth factors may be different between the treated groups, most of them remained on their side of the correlation, with a few exceptions listed in Table 3. When a treatment was added to the culture after inflammation (HA, HAS or the combination of both) MIP- 1α, VEGF, IFNγ, IL-8 and IL-1β behaved differently. HAS supplementation induced changes in BFGF, and HA promoted changes in IL-2, IL-7, IL-13 and IL-12 that were maintained during HAS + HA treatment. Moreover, there were some proteins that positively correlated to both sides of the correlation, acting as switching factors: IL-1β for FCS condition, IL-2 for HA, and IP-10 and IL-2 for HAS + HA treatment (Figure 5E).

## 3. Discussion

This study focuses on the optimization of HAS formulation development, in a way that can ensure scale-up manufacturing as well as the maintenance of the quality of the therapeutic product. For this purpose, primary chondrocytes were kept in culture under different supplementation conditions, including three versions of HAS: one liquid format, also referred in this manuscript as “freshly isolated”, and two lyophilized versions, one of them with an extra IgM filtration step. Moreover, PRP, FCS and combinations of the different HAS formulations in the presence of HA were also compared. It is widely known that lyophilization, or freeze-drying, of sterile therapeutics extends the long-term stability of molecules, including large molecules such as proteins [34,35]. For this reason, lyophilization is currently involved in the manufacturing process of 50% of the total biopharmaceutical products [36,37]. Blood-derived products are usually used in an autologous liquid format and lyophilization may ease the standardization of such products [9]. 

HAS is being tested as a possible therapeutic of OA with the aim of preventing joint degeneration. Previous studies showed that freshly isolated HAS more significantly induced cell proliferation of mesenchymal stromal cells, chondrocytes and in bone explants than PRP supplementation [15,16,17,18]. Moreover, Vacz et al. demonstrated that in ischemia conditions, freshly isolated HAS significantly increased cell proliferation within bone fragments when compared to PRP [14]. Interestingly, previous studies had already shown that PRP increase chondrogenic in vitro proliferation [38,39,40], suggesting that HAS may be a better alternative to induce chondrogenic proliferation when compared to PRP. In this study, a first version of lyophilized HAS, so-called “filtered lyophilized HAS”, did not reach the high supplementation standards of the freshly isolated HAS version and PRP in 2D cultured chondrocytes. We hypothesized that due to the already mentioned extra filtration step during manufacturing, important components involved in promoting cell viability were removed. Therefore, the IgM filtration step was skipped and a new lyophilized HAS, referred as “non-filtered lyophilized HAS”, was produced, which equaled cell viability rates obtained with the freshly isolated formulation. It has been shown that PRP formulations release multiple cytokines in high concentrations, including PDGF-AB, PDGF-BB, transforming growth factor β (TGF- β), VEGF, insulin-like growth factor-1 (IGF-1) and epidermal growth factor (EGF) [3]. Growth factors and protein content of freshly isolated HAS have been analyzed and quantified in previous studies [4,14]. Kuten et al. showed that the in vitro effect of freshly isolated HAS was more predictable and reliable than PRP, probably due to its growth factor profile and the absence of the anticoagulants needed during PRP production. Except IGF-1 release, which had a comparable release from HAS and PRP; VEGF, TGF-β and PDGF presence after HAS supplementation was moderate when compared to PRP [15]. These data support the findings from this study concerning the quantification of VEGF, PDGF-BB and BGFG.

Genes related to joint degeneration and ECM production were quantified. *Col1a1* and *Acan* expressions were increased after supplementation with different formulations of HAS; however, these comparisons were only statistically significant when compared to PRP for *Col1a1*, as previously observed elsewhere [16,17]. Interestingly, *Sox9* and *Col2a1* expression decreased in all conditions after 5 days in culture, which may suggest that cells started to lose the chondrogenic phenotype, as shown in other study where different culture media were used [15]. Results for *Mmp-3*, *Mmp-13* and *Prg4* expression were inconclusive due to the high variability between the donors. In this study, we have not observed a synergistic effect on chondrogenic viability or gene expression of OA-related genes when HAS was supplemented with HA. Contradictory results about similar studies using a combination of PRP and HA can be found in the literature, probably due to the multiple PRP formulations available and the multitude of commercial HA possibilities [33,41,42,43]. Therefore, there is a need for continuing the investigation of the possible synergistic effect of HA and HAS, possibly under mechanical stimulation or in vivo, that are closer to the clinical setting.

In order to better mimic the in vivo OA pathogenesis and to test if non-filtered lyophilized HAS may have a clinical application for it, 3D human explant co-cultures consisting on fragments of bone, articular cartilage and synovium were established and exposed to IL-1β as previously described [19]. It is well known that injured knee joint components can contribute to changes in other joint tissues; this supports the use of in vitro co-culture systems [44,45]. IL-1β is one of the main pro-inflammatory cytokines that plays a role in the pathogenesis of OA; therefore, it is commonly used in vitro for mimicking an OA-diseased state [46,47,48]. Two days after administration of IL-1β, levels of pro-inflammatory cytokines including TNFα, IL-17, IL-2, IL-5 and IL-15; chemokines such as MIP-1α, MIP-1β and IP-10; and the anti-inflammatory cytokines IL-4, IL-10, IL-1Ra and IL-13 increased. As expected, when IL-1β-containing medium was removed, the inflammatory cascade generally diminished. Strikingly, after the therapeutic culture phase, FCS and FCS + HA conditions, but not HAS, induced higher levels of MCP-1, which is a chemoattractant for monocytes and basophils that has been associated with OA [49]. Cell viabilities from the treated tissues were analyzed, and although there was a tendency in the upregulation of cell viability after supplementation with HAS in bone, the level of significance was not reached. However, Kardos et al. demonstrated in a similar experiment with a higher sample size that cell viability was significantly promoted in bone, cartilage and synovium after 5 days of culture in the presence of freshly isolated HAS when compared to FCS [19]. The presence of HA did not alter cell viability of FCS or HAS-supplemented groups. 

Cytokine profiling after treating the inflamed co-cultures with HAS showed a significant decrease of IL-5, IL-15, IL-2, TNFα, IL-7, IL-12 and GM-CSF when compared to FCS. It has been shown that IL-2, IL-12, IL-15, IL-7 and TNFα are upregulated during OA [50,51]. Moreover, the inhibition of GM-CSF revealed pain relief and prevented the development of OA in mice [52], and is therefore a potential target for the treatment of OA and rheumatoid arthritis [53]. Therefore, HAS may be a potential therapeutic to control the inflammation caused during OA. Interestingly, IL-5 is not commonly linked to OA, as its main functions relate to maturation, recruitment and survival of eosinophils. However, a recent study has found that eosinophils abundance is significantly higher at the synovium of OA patients [54]. Moreover, is IL-5 located in the same gene cluster as GM-CSF and IL-4, in chromosome 5q31, which may explain a similar behavior of the cytokines after HAS treatment. In this study, we have also observed that HA-treated co-cultures influenced MIP1-β expression, which, together with IL-2, IL-5 and MCP-1 has been also related severe OA [49]. However, the combination of HAS and HA only reduced the levels of IL-5, suggesting that HAS alone may be more efficient as a treatment for OA; in particular, for the severe stages of the disease. A previous study showed that freshly isolated HAS not only reduced TNFα and IL-12 levels from an explant co-culture, but also IL-1β, IL-6Ra and RANKL. Moreover, it increased MMP-1, MMP-2, MMP-9, COL1A1 and osteonectin, suggesting the activation of tissue remodeling mechanisms [19]. 

Although some of the cytokines and growth factors were not significantly up- or down-regulated, correlations between cytokines were performed. Subgroups after HAS treatment included TNFα, IL-1β and IFNγ, which negative correlated to IL-12 and MIP-1α. The subgroup IL-15, IL-17 and IL-4 was negatively correlated to the cohort containing IL-6, IL-1Ra MIP-1β, IL-7 and IP-10, which, interestingly, has been shown to be a marker for different infections including malaria and dengue fever [55,56]. Finally, the strongly correlated subgroup IL-5, VEGF, MCP-1, IL-8 and the eosinophil chemotactic protein eotaxin was negatively correlated to the cluster formed by IL-2, IL-12, MIP-1α and IL-13, and the first listed cluster. It has been published that the cluster of proteins eotaxin, MCP-1 and VEGF may act as an indicator for osteoarthritic knees; however, MCP-1, VEGF and IL-8 may be a marker for osteoarthritic hips [57]. Moreover, there is evidence that high levels of both IL-12 and IL-13 are related to higher osteoarthritic pain and disability [58]. 

The focus of this study was to develop a standardized HAS product in lyophilized format that reaches the regenerative potential of the previous freshly isolation HAS version and the widely used PRP. The high amount of therapeutics tested in this study restricted most of the experiments conducted to a sample size of 5, which in some cases limited the statistical conclusions where a trend may be observable. Moreover, the HA and HAS synergistic effect could not be proven with the tests performed; further analysis including mechanical stress may be more revealing. It could also be of interest to include more knee joint tissues into our inflammatory co-culture explant model, such as the meniscus, ligaments and infrapatellar fat pad, as well as to study HAS in vivo paracrine effects. To conclude, HAS production has been optimized to obtain a lyophilized formulation that strongly promotes cell viability and has the potential to modulate inflammatory conditions; therefore, it may be a possible therapeutic for OA and joint degeneration.

## 4. Materials and Methods

### 4.1. Primary Osteoarthritic (OA) Chondrocytes Isolation

Human samples from OA patients undergoing complete or partial joint replacement were collected after obtaining a study approval by the Ethics Committee from NiederOesterreich (GS4-EK-4/674-2020) on the 17 November 2020, and prior obtaining a signed informed consent from the patients. Primary chondrocytes were isolated from the remaining articular cartilage of the samples by mincing the cartilage and incubating it for 16 h at 37 °C in serum-free DMEM/F12 GlutaMAX medium (Gibco, 31331093) in the presence of 2 WU liberase (Roche Diagnostics, 5401119001) per gram of cartilage. After the incubation, cell suspension was strained through a 40 µm cell strainer (Corning, 431750), chondrocytes were counted and seeded in culturing DMEM medium supplemented with 10% fetal calf serum (Gibco, 10500064), 200 U/mL Penicillin-0.2 mg/mL Streptomycin (Sigma-Aldrich Chemie GmbH, P4333-100 mL), 2.5 µg/mL Amphotericin B (Sigma-Aldrich Chemie GmbH, A2942-100 mL), and 0.05 mg/mL ascorbic acid (Sigma-Aldrich Chemie GmbH, A5960-25 g). Medium was exchanged weekly during cellular expansion and cells were used when 80% of confluency was reached.

### 4.2. Preparation of PRP 

Whole blood donations from healthy human volunteers between 25 and 45 years old were used to prepare PRP. The exclusion criteria of donors included pregnancy, underweight or diabetes. Whole blood was collected following standards for blood collection and screened for infections. Whole blood was transferred into a citrate-coated tube (VACUETTE 9NC trisodiumcitrate 3.2%, Greiner BioOne, Cat No. 455322) and centrifuged at 440× *g* at room temperature for 10 min. The top fraction, containing the platelets, was transferred to a new tube, and centrifuged again at 1710× *g* at room temperature for 10 min. The platelet pellet was resuspended within one third of the supernatant plasma. PRP from different donors were pooled and stored at −20 °C.

### 4.3. Preparation of Hyperacute Serum (HAS)

Freshly isolated HAS was obtained from whole blood donations from healthy human volunteers, following the already mentioned guidelines. Whole blood was transferred to the HAS Inject Device (OrthoSera) and centrifuged at 1710× *g* at room temperature for 10 min. After centrifugation, the serum and the clot containing red blood cells were discarded. The fibrin clot was isolated and squeezed using flat forceps to obtain the entrapped serum as documented elsewhere [4,14]. Freshly isolated hyperacute sera were pooled and stored at −20 °C. Lyophilized HAS (hypACT, OrthoSera Kft) was prepared from pooled freshly isolated HAS that was filtered with an IgM antibodies BioScale Mini CHT ceramic hydroxyapatite cartridge type II (BioRad, 7324332). Afterwards, 2 mL of pooled sterile serum per vial were frozen at −20 °C for 30 min, and afterwards, cooled with liquid nitrogen for 10 min. Finally, it was freeze dried to produce the lyophilized powder with Labcondo Triad (Fisher Scientific, 794001030). Non-filtered lyophilized HAS (hypACT, OrthoSera Kft) followed the same protocol, excluding the filtration step with the IgM column. Both formulations of lyophilized HAS were stored at room temperature.

### 4.4. Viability Assay of 2D Chondrogenic Cultures

2 × 10^3^ primary isolated chondrocytes per well were seeded in 96-well plates and kept in culture medium (see above) for 48 h. Then, medium was exchanged for different supplemented media and the XTT assay Cell Proliferation kit II (Roche Diagnostics, 11465015001) was performed in triplicates at days 0, 3, 5 and 7. All conditions were prepared from a basal combination of serum-free DMEM medium, Pen/Strep, AmphoB and ascorbic acid as previously described. Then, media were completed as follows: +10% blood-derived product (different formulations of HAS or PRP), +10% FCS, 10% FCS + 2% hyaluronic acid (HA) Monovisc (Anika Therapeutics, 690008), or +10% HAS + 2% HA. Medium was exchanged at day 4.

### 4.5. RNA Isolation and Quantitative Real-Time PCR (qRT-PCR)

A total of 6 × 10^4^ primary isolated chondrocytes per well were seeded in six-well plates and different supplemented media were added (see media conditions above). Total RNA isolation was performed at days 0, 3 and 7 with the High Purity RNA isolation kit (Roche Diagnostics, #11828665001) together with DNAse I (Thermo Scientific, EN0521). Isolated RNA was reverse transcribed with the Transcriptor cDNA synth kit (Roche Diagnostics, 4897030001). For the RT-qPCR quantification, the FastStart Essential DNA Probes Master (Roche Diagnostics, 6402682001) was used. The following genes were analyzed: *Gapdh*, *Acan*, *Col2a1*, *Col1a1*, *Sox9*, *Mmp-3*, *Mmp-13* and *Prg4.* Sequences of primers and probes (3′-5′) can be found in Table 4. Medium was changed at day 4.

### 4.6. Explant Co-Culture for the Inflammatory Model

**Scheme 1:**
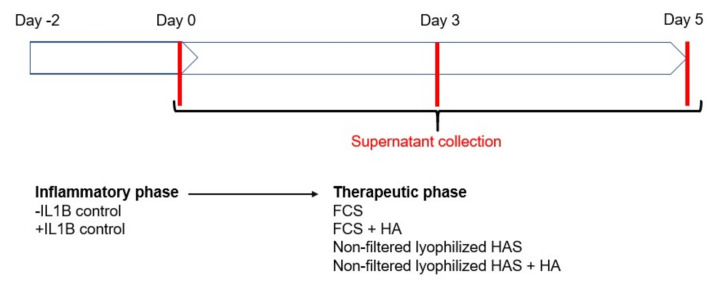
Experimental design of the inflammatory co-culture model experiment. The schematic represents the timeline and the conditioning medium that was used in each phase. Abbreviations: fetal calf serum (FCS), hyperacute serum (HAS), hyaluronic acid (HA), interleukin 1 beta (IL-1β).

Synovial, bone and articular cartilage tissues from human OA samples were identified and minced as explained elsewhere [19]. Five fragments of cartilage, four of bone and two to three of synovium, depending on availability, were placed per well to establish a co-culture. Culture medium was supplemented with 10 ng/mL of IL-1β (Shenandoah, 100-167AF) for 48 h. Then, the inflammatory medium was replaced by different cultured media for 5 days, with medium exchange at day 4. Cultured medium included the following conditions: 10% FCS, 10% FCS + 2% HA, 10% non-filtered lyophilized HAS, 10% non-filtered lyophilized HAS + 2% HA (Scheme 1). Supernatant fluids were collected at days 0, 3 and 5 and proteins were quantified with the Bio-Plex Pro Human Cytokine 27-plex Assay (BioRad, M500KCAF0Y).

### 4.7. Statistical Analysis

GraphPad Prism software (version 7, GraphPad Prism software Inc (Irvine, CA, USA) was used to perform the statistics of this study, where *p* < 0.05 was accepted to determine a statistical significance. For the XTT analysis included in Figure 1, linear regression comparisons were performed. RT-qPCR and XTT data included in Figure 2 and Figure 3 were analyzed via one-way ANOVA test corrected with Tukey’s multiple comparison test. Protein quantifications were analyzed by using one-way ANOVA with Benjamini, Krieger and Yekutieli post-correction for Gaussian distributed samples or Friedman test for non-parametric samples, corrected with Dunn’s multiple comparison test. Pearson correlations were performed and considered strong when r > 0.75. Graphs represent arithmetic mean values ± SD. *p*-values are represented as follows: <0.05 (*) and <0.01 (**).

## Figures and Tables

**Table 1:** Chondrogenic viability after supplementation with hyperacute sera with and without HA.

	HA					
	**−**	**+**	**−**	**+**	**−**	**+**
FCS	1.5722	2.1943	1.9111	1.6714	4.1204	3.8288
Filtered lyophilized HAS	1.8737	1.8371	1.9618	1.0740	3.2265	3.8791
Non-filtered lyo. HAS	2.9374	3.3930	3.2409	3.3741	5.8379	7.0123
	Day 3		Day 5		Day 7	

Mean chondrocyte viability measured by XTT assay at days 3, 5 and 7, comparing filtered and non-filtered lyophilized HAS in the presence or absence of HA. Data represents fold change (in a.u.) normalized to the “d0” condition (n = 5).

**Table 2:** Gene expression of OA-related genes after supplementation with HAS and HA.

	FCS		Filtered Lyophilized		Non-Filtered Lyophilized		
***Genes***	**−**	**+**	**−**	**+**	**−**	**+**	**HA**
***Col1a1***	1.43(±0.82)	1.76(±0.60)	1.55(±1.14)	2.27(±1.21)	2.32(±0.86)	2.45(±0.74)	
***Col2a1***	0.17(±0.12)	0.25(±0.12)	0.15(±0.09)	0.13(±0.12)	0.11(±0.12)	0.12(±0.09)	
***Acan***	2.61(±0.86)	2.70(±0.65)	1.94(±0.76)	2.63(±0.77)	3.77(±2.41)	4.67(±1.98)	
***Mmp-3***	0.16(±0.14)	0.28(±0.26)	0.25(±0.25)	0.33(±0.37)	0.34(±0.47)	0.31(±0.32)	
***Mmp-13***	1.91(±1.59)	1.68(±1.69)	4.44(±5.67)	3.62(±4.31)	6.72(±8.82)	8.85(±13.76)	
***Prg4***	0.30(±0.19)	0.30(±0.23)	0.50(±0.37)	0.49(±0.44)	0.62(±0.62)	0.38(±0.32)	
***Sox9***	0.43(±0.10)	0.38(±0.08)	0.23(±0.04)	0.26(±0.05)	0.22(±0.14)	0.17(±0.07)	

Mean mRNA gene expression (±SD) of OA-related genes from a chondrogenic 2D culture after supplementation with filtered and non-filtered lyophilized HAS in the presence or absence of HA (n = 5).

**Table 3:** List of genes with a differential correlation pattern after treatment supplementation.

Treatment Conditions	Genes
+HAS/+HA/+HAS and +HA	MIP-1α, VEGF, IFNγ, IL-8, IL-1β
+HA	IL-2, IL-7, IL-13, IL-12
+HAS	BFGF
+HAS and +HA	IL-2, IL-7, IP-10, IL-12, IL-13, MIP-1β

**Table 4:** List of sequences for primers and probes used during gene expression analysis (3′-5′).

Gene	Forward Primer	Reverse Primer	Probe
*Gapdh*	ATGTTCCAGTATGATTCCACCC	ATACTCAGCACCAGCATCAC	AGCTTCCCGTTCTCTGCCTTGAC
*Acan*	ACCTACGATGTCTACTGCTACG	AGAGTGGCGTTTTGGGATTC	AGAAGGTGAACTGCTCCAGGCG
*Col2a1*	GTGCAACTGGTCCTCTGG	ACCTCTTTTCCCTTCTTCACC	CCTTGTTCGCCTTTGAAGCCAGC
*Col1a1*	CCCCTGGAAAGAATGGAGATG	TCCAAACCACTGAAACCTCTG	TTCCGGGCAATCCTCGAGCA
*Sox9*	ACTTGCACAACGCCGAG	CTGGTACTTGTAATCCGGGTG	TCTGGAGACTTCTGAACGAGAGCGA
*Mmp-3*	CCAGGGATTAATGGAGATGCC	AGTGTTGGCTGAGTGAAAGAG	ACAATGGACAAAGGATACAACAGGGACC
*Mmp-13*	CTAAACATCCCAAAACGCCAG	ACAGCTCTGCTTCAACCTG	CCCTTGATGCCATAACCAGTCTCCG
*Prg4*	AGAACTGGCCTGAATCTGTG	ACCTGTGTCGTTTCTCCATAC	TCAAGAGAGGTGGCAGCATTCAGC

## Data Availability

The data presented in this study are openly available in FigShare, doi:10.6084/m9.figshare.14973663.

## Supplementary Materials

The following are available online at https://www.mdpi.com/article/10.3390/ijms22147496/s1, Table S1: List of Pearson correlation values for the different conditions, Table S2: List of Pearson correlation values for the different conditions.
