# Supplementary material for: Optimization of Lyophilized Hyperacute Serum (HAS) as a Regenerative Therapeutic in Osteoarthritis
Source: Int J Mol Sci. 2021 Jul 13;22(14):7496. doi: 10.3390/ijms22147496 (PMC8305834; doi:10.3390/ijms22147496)
Supplement: Supplementary file 1 [file ijms-22-07496-s001.zip › ijms-1276630-supplementary.pdf]

Table S1: Complete list of p-values of the supernatant fluids analysis corresponding to Figure 4. Inflamed control (IC).

	FCS vs FCS+HA	FCS vs HAS	HAS vs HAS+HA	FCS+HA vs HAS+HA	IC vs FCS	IC vs FCS+HA	IC vs HAS	IC vs HAS+HA
PDGF-BB	>0.999	0.231	>0.999	0.231	0.080	0.250	>0.999	0.485
IL-12	0.196	0.048	0.525	0.062	0.331	0.331	0.331	0.331
MIP-1β	0.045	0.100	0.291	0.881	>0.999	0.018	0.282	0.080
IL-1Ra	0.258	0.082	0.464	0.540	0.252	0.070	0.252	0.252
IL-6	>0.999	>0.999	>0.999	>0.999	>0.999	>0.999	0.485	0.786
GM-CSF	>0.999	0.045	>0.999	0.823	>0.999	0.485	0.018	0.039
RANTES	>0.999	0.107	>0.999	0.455	>0.999	0.786	>0.999	>0.999
IL-2	0.214	0.034	0.719	0.419	>0.999	0.373	0.036	0.080
IL-9	0.939	0.242	0.482	0.217	0.282	0.155	>0.999	>0.999
IL-10	0.373	0.622	>0.999	>0.999	0.009	0.009	0.009	0.009
G-CSF	>0.999	0.107	>0.999	0.455	>0.999	>0.999	0.203	0.080
IP-10	0.378	0.376	0.606	0.546	0.263	0.263	0.263	0.263
IL-13	0.241	0.791	0.936	0.098	0.282	0.157	0.485	0.786
VEGF	0.413	0.103	0.233	0.135	>0.999	>0.999	0.282	>0.999
IL-7	0.647	0.031	0.968	0.212	0.320	0.320	0.214	0.320
IFNγ	0.823	>0.999	>0.999	>0.999	>0.999	0.373	0.056	0.056
IL-8	0.311	0.142	0.173	0.401	>0.999	>0.999	0.786	>0.999
IL-1β	>0.999	>0.999	>0.999	>0.999	0.485	0.080	0.282	0.155
MIP-1α	>0.999	0.786	>0.999	>0.999	>0.999	0.282	0.080	0.039
IL-15	0.994	0.045	0.173	0.065	0.122	0.122	0.122	0.122
MCP-1	>0.999	0.786	>0.999	0.065	0.018	0.039	0.485	>0.999
BFGF	0.399	0.400	0.526	0.320	0.308	0.308	0.308	0.308
IL-5	0.517	0.021	0.021	0.321	0.101	0.101	0.018	0.024
IL-4	>0.999	0.786	>0.999	>0.999	0.786	0.485	0.039	0.080
IL-17	>0.999	0.981	>0.999	>0.999	>0.999	0.211	0.112	0.039
TNFα	0.282	0.038	>0.999	>0.999	>0.999	0.039	0.080	0.155
Eotaxin	0.566	0.304	0.894	0.092	>0.999	>0.999	>0.999	0.786

Table S2: List of Pearson correlation values for the different conditions

FCS

	PDGF-BB	IL-12	MIP-1β	IL-1ra	IL-6	GM-CSF	RANTES	IL-2	IL-9	IL-10	G-CSF	IP-10	IL-13	VEGF	IL-7	IFNγ	IL-8	IL-1β	MIP-1α	IL-15	MCP-1	BFGF	IL-5	IL-4	IL-17	TNFα	Eotaxin
PDGF-BB	1,000																										
IL-12	0,950	1,000																									
MIP-1β	0,902	0,992	1,000																								
IL-1ra	0,914	0,995	1,000	1,000																							
IL-6	0,786	0,940	0,976	0,969	1,000																						
GM-CSF	0,619	0,833	0,897	0,884	0,972	1,000																					
RANTES	0,535	0,772	0,847	0,831	0,943	0,995	1,000																				
IL-2	0,520	0,761	0,838	0,821	0,937	0,993	1,000	1,000																			
IL-9	0,453	0,709	0,793	0,775	0,908	0,981	0,996	0,997	1,000																		
IL-10	0,484	0,733	0,814	0,797	0,922	0,987	0,998	0,999	0,999	1,000																	
G-CSF	0,505	0,749	0,828	0,811	0,931	0,990	0,999	1,000	0,998	1,000	1,000																
IP-10	0,651	0,856	0,915	0,903	0,981	0,999	0,990	0,987	0,972	0,979	0,984	1,000															
IL-13	0,283	0,568	0,669	0,647	0,816	0,928	0,962	0,966	0,983	0,976	0,971	0,912	1,000														
VEGF	0,981	0,871	0,801	0,818	0,651	0,455	0,361	0,345	0,272	0,305	0,328	0,491	0,091	1,000													
IL-7	0,971	0,847	0,772	0,790	0,614	0,411	0,316	0,299	0,225	0,259	0,282	0,449	0,043	0,999	1,000												
IFNγ	0,939	0,785	0,699	0,720	0,525	0,311	0,212	0,195	0,119	0,154	0,177	0,351	-0,064	0,988	0,994	1,000											
IL-8	0,802	0,575	0,466	0,491	0,260	0,027	-0,075	-0,093	-0,169	-0,134	-0,111	0,069	-0,346	0,903	0,922	0,958	1,000										
IL-1β	0,582	0,299	0,175	0,203	-0,046	-0,279	-0,375	-0,392	-0,461	-0,430	-0,408	-0,238	-0,615	0,729	0,761	0,826	0,953	1,000									
MIP-1α	-0,972	-0,997	-0,979	-0,984	-0,910	-0,787	-0,720	-0,708	-0,651	-0,678	-0,695	-0,812	-0,502	-0,907	-0,886	-0,831	-0,638	-0,373	1,000								
IL-15	-0,999	-0,960	-0,916	-0,927	-0,806	-0,645	-0,563	-0,548	-0,483	-0,513	-0,533	-0,676	-0,315	-0,974	-0,962	-0,927	-0,782	-0,555	0,979	1,000							
MCP-1	-0,730	-0,907	-0,953	-0,944	-0,996	-0,989	-0,968	-0,963	-0,940	-0,951	-0,958	-0,994	-0,862	-0,584	-0,544	-0,451	-0,177	0,131	0,871	0,752	1,000						
BFGF	-0,755	-0,922	-0,964	-0,956	-0,999	-0,982	-0,958	-0,953	-0,927	-0,939	-0,947	-0,989	-0,842	-0,614	-0,575	-0,484	-0,214	0,093	0,889	0,777	0,999	1,000					
IL-5	-0,340	-0,617	-0,712	-0,692	-0,849	-0,949	-0,976	-0,980	-0,992	-0,987	-0,984	-0,935	-0,998	-0,151	-0,104	0,004	0,289	0,567	0,553	0,371	0,891	0,873	1,000				
IL-4	-0,224	-0,517	-0,622	-0,599	-0,779	-0,904	-0,943	-0,949	-0,970	-0,961	-0,954	-0,885	-0,998	-0,030	0,017	0,125	0,403	0,662	0,448	0,256	0,829	0,808	0,993	1,000			
IL-17	0,052	-0,262	-0,383	-0,356	-0,577	-0,752	-0,815	-0,825	-0,866	-0,848	-0,836	-0,724	-0,943	0,245	0,291	0,392	0,639	0,843	0,185	-0,019	0,644	0,615	0,921	0,962	1,000		
TNFα	0,146	-0,170	-0,294	-0,266	-0,497	-0,686	-0,757	-0,768	-0,815	-0,794	-0,780	-0,655	-0,907	0,336	0,380	0,478	0,708	0,890	0,092	-0,114	0,569	0,538	0,880	0,931	0,996	1,000	
Eotaxin	0,104	-0,212	-0,334	-0,307	-0,533	-0,717	-0,784	-0,795	-0,839	-0,819	-0,806	-0,687	-0,924	0,296	0,341	0,440	0,678	0,870	0,134	-0,071	0,603	0,573	0,900	0,946	0,999	0,999	1,000

FCS + HA

	MIP-1β	IL-6	IL-1ra	GM-CSF	IL-10	RANTES	IL-9	G-CSF	PDGF-BB	MIP-1α	IL-2	IL-1β	Eotaxin	MCP-1	IFNγ	IP-10	BFGF	IL-4	IL-15	IL-13	IL-8	IL-17	IL-12	IL-7	TNFα	IL-5	VEGF
MIP-1β	1,000																										
IL-6	0,995	1,000																									
IL-1ra	0,995	1,000	1,000																								
GM-CSF	0,999	0,998	0,998	1,000																							
IL-10	0,987	0,998	0,998	0,992	1,000																						
RANTES	0,973	0,991	0,991	0,981	0,997	1,000																					
IL-9	0,863	0,908	0,908	0,881	0,933	0,956	1,000																				
G-CSF	0,813	0,866	0,866	0,834	0,896	0,926	0,996	1,000																			
PDGF-BB	0,516	0,598	0,598	0,549	0,648	0,701	0,879	0,919	1,000																		
MIP-1α	0,226	0,320	0,320	0,263	0,380	0,446	0,688	0,751	0,951	1,000																	
IL-2	-0,058	0,040	0,040	-0,020	0,103	0,175	0,455	0,534	0,825	0,959	1,000																
IL-1β	-0,260	-0,353	-0,353	-0,297	-0,412	-0,477	-0,712	-0,774	-0,961	-0,999	-0,949	1,000															
Eotaxin	-0,643	-0,715	-0,715	-0,672	-0,758	-0,803	-0,942	-0,969	-0,988	-0,891	-0,727	0,907	1,000														
MCP-1	-0,497	-0,580	-0,580	-0,530	-0,630	-0,685	-0,868	-0,910	-1,000	-0,958	-0,837	0,967	0,984	1,000													
IFNγ	-0,334	-0,425	-0,425	-0,371	-0,482	-0,544	-0,765	-0,821	-0,980	-0,994	-0,921	0,997	0,937	0,984	1,000												
IP-10	0,716	0,644	0,644	0,688	0,594	0,534	0,264	0,174	-0,229	-0,519	-0,739	0,489	0,075	0,250	0,419	1,000											
BFGF	-0,566	-0,483	-0,483	-0,534	-0,426	-0,360	-0,072	0,020	0,413	0,675	0,856	-0,649	-0,267	-0,434	-0,587	-0,981	1,000										
IL-4	-0,658	-0,581	-0,581	-0,628	-0,528	-0,465	-0,187	-0,096	0,305	0,585	0,790	-0,556	-0,154	-0,327	-0,490	-0,997	0,993	1,000									
IL-15	-0,546	-0,462	-0,461	-0,514	-0,404	-0,337	-0,047	0,044	0,435	0,693	0,868	-0,667	-0,290	-0,455	-0,607	-0,976	1,000	0,990	1,000								
IL-13	-0,973	-0,946	-0,945	-0,963	-0,923	-0,893	-0,722	-0,656	-0,304	0,006	0,288	0,029	0,448	0,283	0,107	-0,858	0,742	0,814	0,725	1,000							
IL-8	-0,980	-0,956	-0,956	-0,972	-0,936	-0,908	-0,746	-0,682	-0,338	-0,030	0,254	0,065	0,480	0,317	0,143	-0,839	0,717	0,793	0,700	0,999	1,000						
IL-17	-0,899	-0,852	-0,852	-0,882	-0,817	-0,773	-0,555	-0,476	-0,090	0,223	0,489	-0,189	0,243	0,067	-0,111	-0,949	0,870	0,921	0,857	0,976	0,968	1,000					
IL-12	-0,985	-0,963	-0,963	-0,978	-0,944	-0,918	-0,763	-0,700	-0,361	-0,055	0,229	0,090	0,502	0,340	0,167	-0,825	0,700	0,778	0,682	0,998	1,000	0,961	1,000				
IL-7	-0,890	-0,930	-0,930	-0,907	-0,952	-0,972	-0,998	-0,989	-0,850	-0,645	-0,403	0,671	0,921	0,838	0,727	-0,319	0,128	0,242	0,104	0,760	0,783	0,601	0,798	1,000			
TNFα	-0,879	-0,921	-0,921	-0,896	-0,944	-0,965	-0,999	-0,992	-0,863	-0,664	-0,426	0,689	0,931	0,851	0,744	-0,295	0,104	0,218	0,080	0,744	0,767	0,581	0,783	1,000	1,000		
IL-5	-0,999	-0,998	-0,998	-1,000	-0,992	-0,981	-0,880	-0,833	-0,547	-0,261	0,022	0,295	0,671	0,528	0,369	-0,690	0,536	0,630	0,515	0,964	0,973	0,883	0,978	0,906	0,895	1,000	
VEGF	-1,000	-0,993	-0,993	-0,998	-0,983	-0,967	-0,851	-0,800	-0,498	-0,205	0,080	0,239	0,626	0,478	0,314	-0,731	0,584	0,674	0,564	0,978	0,985	0,909	0,989	0,880	0,868	0,998	1,000

HAS

	MIP-1β	IL-1ra	IL-7	IL-6	IP-10	GM-CSF	IL-10	G-CSF	RANTES	IL-9	IL-2	MIP-1α	IL-12	BFGF	IL-13	PDGF-BB	Eotaxin	IL-5	MCP-1	VEGF	IL-8	IL-4	IL-15	IL-17	TNFα	IL-1β	IFNγ
MIP-1β	1,000																										
IL-1ra	1,000	1,000																									
IL-7	0,984	0,984	1,000																								
IL-6	0,968	0,968	0,997	1,000																							
IP-10	0,889	0,889	0,957	0,976	1,000																						
GM-CSF	0,944	0,944	0,869	0,830	0,688	1,000																					
IL-10	0,886	0,886	0,788	0,741	0,575	0,989	1,000																				
G-CSF	0,867	0,867	0,763	0,714	0,542	0,983	0,999	1,000																			
RANTES	0,765	0,765	0,637	0,579	0,385	0,935	0,976	0,984	1,000																		
IL-9	0,661	0,661	0,515	0,451	0,244	0,872	0,934	0,947	0,989	1,000																	
IL-2	0,569	0,569	0,411	0,344	0,129	0,809	0,885	0,903	0,965	0,993	1,000																
MIP-1α	0,325	0,325	0,149	0,077	-0,144	0,619	0,726	0,753	0,858	0,924	0,963	1,000															
IL-12	0,409	0,408	0,237	0,166	-0,055	0,687	0,785	0,809	0,900	0,955	0,983	0,996	1,000														
BFGF	-0,057	-0,057	-0,236	-0,306	-0,508	0,276	0,412	0,449	0,599	0,712	0,789	0,926	0,888	1,000													
IL-13	-0,027	-0,027	-0,207	-0,278	-0,482	0,305	0,439	0,475	0,623	0,732	0,807	0,937	0,901	1,000	1,000												
PDGF-BB	-0,440	-0,440	-0,595	-0,652	-0,803	-0,119	0,026	0,066	0,241	0,383	0,488	0,706	0,640	0,921	0,910	1,000											
Eotaxin	-0,733	-0,732	-0,598	-0,538	-0,339	-0,916	-0,965	-0,974	-0,999	-0,995	-0,977	-0,882	-0,921	-0,638	-0,661	-0,289	1,000										
IL-5	-0,670	-0,670	-0,525	-0,462	-0,255	-0,878	-0,938	-0,951	-0,991	-1,000	-0,992	-0,920	-0,951	-0,703	-0,724	-0,372	0,996	1,000									
MCP-1	-0,903	-0,903	-0,811	-0,766	-0,606	-0,994	-0,999	-0,997	-0,967	-0,919	-0,867	-0,700	-0,761	-0,377	-0,405	0,012	0,954	0,924	1,000								
VEGF	-0,994	-0,994	-0,957	-0,933	-0,831	-0,975	-0,933	-0,918	-0,833	-0,742	-0,658	-0,430	-0,509	-0,057	-0,086	0,336	0,805	0,749	0,946	1,000							
IL-8	-0,984	-0,984	-0,936	-0,908	-0,794	-0,987	-0,954	-0,941	-0,867	-0,784	-0,705	-0,487	-0,564	-0,121	-0,150	0,274	0,841	0,791	0,965	0,998	1,000						
IL-4	-0,754	-0,754	-0,860	-0,895	-0,971	-0,495	-0,364	-0,326	-0,154	-0,006	0,111	0,376	0,291	0,699	0,677	0,922	0,105	0,017	0,399	0,675	0,626	1,000					
IL-15	-0,856	-0,856	-0,935	-0,958	-0,998	-0,637	-0,519	-0,484	-0,322	-0,178	-0,061	0,211	0,122	0,565	0,540	0,841	0,275	0,189	0,551	0,792	0,751	0,985	1,000				
IL-17	-0,899	-0,900	-0,964	-0,980	-1,000	-0,705	-0,595	-0,562	-0,407	-0,267	-0,152	0,121	0,031	0,487	0,461	0,788	0,361	0,278	0,625	0,844	0,808	0,965	0,996	1,000			
TNFα	-0,089	-0,088	0,092	0,165	0,377	-0,413	-0,540	-0,573	-0,709	-0,806	-0,870	-0,971	-0,945	-0,989	-0,993	-0,855	0,743	0,799	0,508	0,201	0,264	-0,587	-0,440	-0,356	1,000		
IL-1β	0,064	0,065	0,243	0,313	0,514	-0,269	-0,405	-0,442	-0,593	-0,706	-0,784	-0,923	-0,884	-1,000	-0,999	-0,924	0,632	0,698	0,370	0,049	0,113	-0,704	-0,572	-0,494	0,988	1,000	
IFNγ	0,173	0,173	0,348	0,415	0,605	-0,162	-0,303	-0,341	-0,502	-0,625	-0,712	-0,875	-0,828	-0,993	-0,989	-0,961	0,544	0,616	0,267	-0,060	0,004	-0,777	-0,658	-0,586	0,966	0,994	1,000

HAS+ HA

	MIP-1β	IL-1β	IFNγ	IL-8	IL-1ra	IL-10	G-CSF	GM-CSF	RANTES	MIP-1α	IL-9	IL-6	PDGF-BB	IL-12	BFGF	VEGF	IL-5	IL-7	IL-13	IL-4	Eotaxin	IL-15	IL-17	MCP-1	TNFα	IL-2	IP-10
MIP-1β	1,000																										
IL-1β	0,934	1,000																									
IFNγ	0,923	1,000	1,000																								
IL-8	0,997	0,905	0,892	1,000																							
IL-1ra	0,536	0,199	0,170	0,596	1,000																						
IL-10	-0,039	-0,394	-0,421	0,034	0,823	1,000																					
G-CSF	-0,058	-0,412	-0,439	0,015	0,811	1,000	1,000																				
GM-CSF	0,105	-0,258	-0,287	0,177	0,896	0,990	0,987	1,000																			
RANTES	-0,334	-0,649	-0,671	-0,264	0,617	0,955	0,960	0,902	1,000																		
MIP-1α	-0,559	-0,819	-0,836	-0,497	0,400	0,850	0,860	0,766	0,968	1,000																	
IL-9	-0,662	-0,886	-0,900	-0,605	0,278	0,775	0,787	0,676	0,928	0,992	1,000																
IL-6	-0,800	-0,962	-0,969	-0,754	0,078	0,631	0,646	0,513	0,833	0,945	0,979	1,000															
PDGF-BB	-0,905	-0,997	-0,999	-0,871	-0,126	0,461	0,478	0,329	0,704	0,859	0,918	0,979	1,000														
IL-12	-0,439	-0,089	-0,059	-0,504	-0,994	-0,880	-0,871	-0,939	-0,700	-0,499	-0,383	-0,188	0,015	1,000													
BFGF	-0,226	0,138	0,167	-0,296	-0,943	-0,965	-0,959	-0,992	-0,843	-0,681	-0,581	-0,404	-0,211	0,974	1,000												
VEGF	-0,146	0,218	0,247	-0,218	-0,913	-0,983	-0,979	-0,999	-0,884	-0,738	-0,645	-0,477	-0,289	0,953	0,997	1,000											
IL-5	-0,320	0,040	0,069	-0,389	-0,971	-0,934	-0,927	-0,976	-0,786	-0,606	-0,498	-0,312	-0,114	0,992	0,995	0,984	1,000										
IL-7	0,099	0,448	0,475	0,026	-0,787	-0,998	-0,999	-0,979	-0,971	-0,880	-0,811	-0,676	-0,513	0,851	0,947	0,970	0,911	1,000									
IL-13	0,264	0,592	0,615	0,193	-0,673	-0,974	-0,978	-0,932	-0,997	-0,947	-0,898	-0,790	-0,649	0,751	0,880	0,916	0,829	0,986	1,000								
IL-4	0,184	0,524	0,549	0,112	-0,731	-0,989	-0,992	-0,958	-0,988	-0,918	-0,859	-0,737	-0,585	0,802	0,916	0,945	0,872	0,996	0,997	1,000							
Eotaxin	0,417	0,715	0,735	0,350	-0,543	-0,924	-0,931	-0,860	-0,996	-0,987	-0,958	-0,879	-0,765	0,633	0,791	0,838	0,727	0,945	0,987	0,970	1,000						
IL-15	0,439	0,731	0,751	0,372	-0,523	-0,915	-0,923	-0,848	-0,994	-0,990	-0,964	-0,890	-0,780	0,614	0,776	0,825	0,710	0,937	0,982	0,964	1,000	1,000					
IL-17	0,382	0,687	0,709	0,313	-0,575	-0,938	-0,945	-0,879	-0,999	-0,980	-0,946	-0,860	-0,739	0,662	0,814	0,859	0,753	0,957	0,992	0,979	0,999	0,998	1,000				
MCP-1	0,710	0,915	0,927	0,657	-0,213	-0,731	-0,744	-0,626	-0,901	-0,981	-0,998	-0,991	-0,942	0,320	0,525	0,593	0,439	0,771	0,866	0,823	0,936	0,944	0,922	1,000			
TNFα	0,737	0,930	0,941	0,686	-0,175	-0,704	-0,717	-0,595	-0,883	-0,972	-0,994	-0,995	-0,955	0,283	0,492	0,561	0,404	0,745	0,846	0,800	0,922	0,931	0,906	0,999	1,000		
IL-2	0,791	0,520	0,495	0,834	0,940	0,580	0,564	0,691	0,312	0,064	-0,065	-0,266	-0,456	-0,897	-0,774	-0,720	-0,833	-0,530	-0,381	-0,455	-0,225	-0,202	-0,263	0,132	0,171	1,000	
IP-10	0,651	0,336	0,308	0,705	0,990	0,733	0,720	0,823	0,498	0,265	0,138	-0,065	-0,266	-0,968	-0,886	-0,846	-0,928	-0,691	-0,560	-0,626	-0,418	-0,396	-0,453	-0,072	-0,033	0,979	1,000
